# Theta oscillation and neuronal activity in rat hippocampus are involved in temporal discrimination of time in seconds

**DOI:** 10.3389/fnsys.2015.00095

**Published:** 2015-06-22

**Authors:** Tomoaki Nakazono, Tomomi Sano, Susumu Takahashi, Yoshio Sakurai

**Affiliations:** ^1^Department of Psychology, Graduate School of Letters, Kyoto UniversityKyoto, Japan; ^2^Laboratory of Neural Information, Graduate School of Brain Science, Doshisha UniversityKyotanabe, Japan; ^3^Laboratory of Neural Circuitry, Graduate School of Brain Science, Doshisha UniversityKyotanabe, Japan

**Keywords:** time perception, time cell, theta oscillation, hippocampus, duration discrimination

## Abstract

The discovery of time cells revealed that the rodent hippocampus has information regarding time. Previous studies have suggested that the role of hippocampal time cells is to integrate temporally segregated events into a sequence using working memory with time perception. However, it is unclear whether hippocampal cells contribute to time perception itself because most previous studies employed delayed matching-to-sample tasks that did not separately evaluate time perception from working memory processes. Here, we investigated the function of the rat hippocampus in time perception using a temporal discrimination task. In the task, rats had to discriminate between durations of 1 and 3 s to get a reward, and maintaining task-related information as working memory was not required. We found that some hippocampal neurons showed firing rate modulation similar to that of time cells. Moreover, theta oscillation of local field potentials (LFPs) showed a transient enhancement of power during time discrimination periods. However, there were little relationships between the neuronal activities and theta oscillations. These results suggest that both the individual neuronal activities and theta oscillations of LFPs in the hippocampus have a possibility to be engaged in seconds order time perception; however, they participate in different ways.

## Introduction

Time perception is an important cognitive function for animals. However, the neural mechanisms underlying this perception are still unclear. Particularly, the mechanisms of time perception in interval timing (i.e., seconds-to-minutes time scale), is unknown, although it is thought to be an important basis of conscious time estimation (Buhusi and Meck, 2005). Some brain components, such as corticostriatal circuits and dopaminergic neurons, have been reported to be involved in interval timing (Buhusi and Meck, 2005); however, other brain structures and their neural activities should be investigated.

Recently, the role of the hippocampus in interval timing has received attention. Because the hippocampus has been considered to be an important region for episodic memory, previous studies have focused on its contribution to temporal organization of memories. Naya and Suzuki (2011) demonstrated that hippocampal neurons in monkeys provide timing signals that organize the temporal order of events. In rodents, multiple studies have reported that neurons in the hippocampal CA1 region sequentially fired as if they maintained memories or represented elapsed time (Pastalkova et al., 2008; Gill et al., 2011; MacDonald et al., 2011, 2013; Kraus et al., 2013; Modi et al., 2014). Furthermore, MacDonald et al. (2011) reported that the properties of the neurons was similar to that of place cells (O’Keefe and Dostrovsky, 1971) and thus named them time cells. Time cells have also been observed in animals that were immobilized (MacDonald et al., 2013).

Despite these findings, the role of the hippocampus in time perception and interval timing remains unclear. A previous study reported that lesions of the hippocampus changed time perception (Meck et al., 1984). Conversely, recent lesion studies (Jackson et al., 1998; Kyd et al., 2008) and studies using pharmacological inactivation (Jacobs et al., 2013) have reported that the hippocampus is unnecessary for interval timing. These studies suggested that the role of time cells is to temporally organize segregated events into a sequence, rather than time perception itself (Jacobs et al., 2013). This hypothesis is consistent with the result of a study on time cells. Pastalkova et al. (2008) reported that sequential firing in the hippocampus does not occur in non-memory conditions.

Jacobs et al. (2013) reported that performance on an interval discrimination of the time in seconds was facilitated by hippocampal inactivation, and they hypothesized that the hippocampus competitively interacts with another timing system during perception of the short timescales. Such competitive interaction may require the mechanism to communicate between the hippocampus and other time-processing systems. We hypothesize that local field potentials (LFPs), particularly theta oscillation, are candidates for such interaction mechanisms because LFPs are believed to have roles in interactions between the brain areas (Jones and Wilson, 2005; van der Meer and Redish, 2011; Terada et al., 2013). Additionally, some studies have suggested that hippocampal theta oscillation may participate in interval timing (Onoda et al., 2003; Hattori and Sakata, 2014).

In this study, we designed a temporal discrimination task that does not require working memory to study active time perception, specifically interval discrimination. We recorded the neuronal activities in the hippocampus of rats while they performed this discrimination task. These experiments allowed us to investigate the role of hippocampal LFPs, and the relationship between LFPs and other neuronal activities in the time perception of interval timing.

## Materials and Methods

### Subjects

Eight male Wistar albino rats (Shimizu Laboratory Supplies, Kyoto, Japan), each weighing 480–580 g at the time of the experiment and housed in a 25 cm × 15 cm × 24 cm cage, were used as experimental subjects. Data from four rats (#4, #5, #7, and #8 in **Table 1**) were used only for LFP analysis, and data from one rat (#6) was used only for neuronal analysis. All rats were extensively handled, provided with a sufficient amount of lab chow 1–3 h after each daily training or recording session to maintain ∼80–90% of their ad libitum weight and allowed free access to water. They were exposed to light between 08:00 and 21:00 h each day. All experimental procedures were in accord with the guidelines presented in the Guidelines for Care and Use of Laboratory Animals at Kyoto University (2007) and had the approval of the Animal Research Committee of Kyoto University.

**Table 1:** Reaction time (RT) and enhancement of theta oscillation data.

Reaction time (ms)				Enhancement of theta (ms)				
Rat (Color)	RT (Long)	RT (Short)	*p* value	Start	End	Duration	Peak	Peak (Control)
1 (yellow)	1766.9	1658.4	0.050	400	1200	800	800	
2 (green)	1465.8	1305.5	<0.001^∗^	0	2350	2350	750	
3 (cyan)	1453.2	1499.5	0.955	450	1150	700	700	
4 (magenta)	2144.6	1201.4	0.148	800	1350	550	1100	
5 (blue)	1498.4	1451.3	0.383	450	850	400	750	2650
6	1292.6	1274.9	0.054					
7 (red)	618.3	717.4	<0.001 ^∗^	200	900	700	550	2750
8 (black)	635.0	684.5	0.056	550	2550	2000	1450	2350

*Rat (Color) corresponds to the color used to plot the data of the rat in **Figures 4C** and **5A** RT (Long) and RT (Short) are medians of RT. p value shows the results of the Mann–Whitney U-test between RTs in the short and long fixation trials for each rat. Asterisks indicate significant differences of p < 0.05. Enhancement of theta shows start timings (Start), end timings (End), durations (Duration), peak timings (Peak), and peak timings in control experiment [Peak (Control)] of the theta enhancement period (see section 3.3.). Each timing shows the time from the start of the fixation period. We did not use LFP data of rat #6 for analysis because of the noise of the data. We performed a control experiment with three animals (#5, #7, and #8)*.

### Apparatus

In a dim, sound-attenuated, electrically shielded box (Japan Shield Enclosure, Osaka, Japan), which included a 175 mm × 320 mm × 450 mm operant chamber (OharaIka, Tokyo, Japan), rats were trained on a behavioral task. One wall of the chamber had three illuminated sensor holes, which were used to detect the nose-poke behavior of rats; the sensors were 15 mm in diameter and horizontally arranged 60 mm above the floor. Access to the left and right holes was controlled using a guillotine door immediately in front of each hole. A food dispenser located behind the wall delivered 25-mg food pellets to a food magazine located in the center of the wall and 10 mm above the floor. The dispenser delivered pellets coupled with an intermittent low buzzer tone (reward tone). Another buzzer was located 400 mm above the floor of the wall and delivered a continuous buzzer tone when the rats made erroneous responses (error tone). In a control experiment, additional visual stimuli were presented on the left or right wall using a light-emitting diode (LED). The task was controlled and behavioral data were recorded using a personal computer (NEC, Tokyo, Japan).

### Behavioral Task

At the first of behavioral training, the rats were trained to continue nose poking for 3 s (shaping). In the shaping process, the rats poked their noses in the central hole, and continued to do so until the guillotine door opened, which occurred after particular period. After the guillotine door opened, one of two more holes, a left and right hole, became illuminated and accessible for 3 s. The rats should choose the illuminated hole in order to get a food reward. Shaping was started with 100 ms of waiting period, and if the animals could continue nose-poking successfully, the period was extended step by step. After the period reached 3 s, shaping was finished.

**FIGURE 1:**
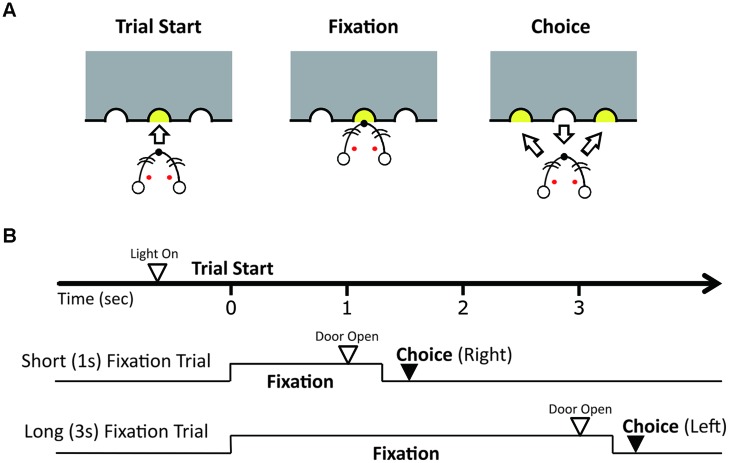
**Duration discrimination task**. **(A)** Illustration of the discrimination trial. The rat pokes its nose into the central hole to start a trial and fixates its nose in the hole until the guillotine door opens and the lights at the side holes turn on. Fixation periods were either 1 or 3 s. After the door opens, the rat had to choose the right hole when the fixation period was 1 s and the left hole when the fixation period was 3 s to receive a reward. **(B)** Illustration of the sequence of events and correct responses in trials with the short and long fixation periods.

After the shaping, each rat was trained for a duration discrimination task (**Figure 1**) until they reached a rate of 70% correct trials per session. There was an inter-trial interval (ITI) of 5 s. Before each trial, the guillotine door was closed and only a central hole was accessible. Rats poked their noses in the central hole, and continued to do so until the guillotine door opened, which occurred after 1 s (short fixation trials) or 3 s (long fixation trials) from the time they first poked their nose in the hole. We called the periods of continued nose-poking as fixation periods. The short and long fixation periods were presented in a random order. After the guillotine door opened, two more holes, a left and right hole, became illuminated and accessible for 3 s. The rats learned to choose the right hole in the short fixation trials and the left hole in long fixation trials in order to get a food reward. If the rat removed their nose from the central hole for longer than 300 ms before the door opened, the trial was scored as an incorrect response. An incorrect response closed the door, produced a 1-s buzzer noise, and was followed by a series of correction trials until the rat chose the correct hole from which no data were counted. Each training or recording session continued for a maximum of 160 trials, excluding correction trials, or until 90 min had elapsed.

A control experiment was performed with three rats (#5, #7, and #8) to examine the effect of nose-poking movements. In this experiment, there was a fixation period of 3 s only, and either the left or right wall’s LED and hole were illuminated after a 3-s fixation period. The rat learned to choose the hole on the illuminated side to get a reward. After the recording session of the experimental condition, three rats were trained in the control experiment until they achieved a rate of 80% or more correct trials per session.

### Electrode Construction, Implantation, and Recording

Neuronal recording was conducted with two bundles of five tungsten microwires (12.5 micron in diameter; California Fine Wire, Grover Beach, CA, USA). In each bundle, one of the five microwires was branched away and used for LFP recording, and the other four wires were used as a tetrode (Wilson and McNaughton, 1993) to record multi-neuronal activities. The five microwires were mounted in a 33-gage stainless-steel cannula (Small Parts, Miami, FL, USA) with 500 microns of the tip protruding. The tips were cut at right angles with sharp surgical scissors. The tip impedance was ∼400 kΩ (at 1 kHz). Cannulas were attached in a row to construct an array of tetrodes, with a center-to-center spacing of 500 microns between the cannulas. The array of tetrodes was mounted on a microdrive assembly (McNaughton et al., 1989; Sakurai, 2002; Sakurai and Takahashi, 2006; Terada et al., 2013) that was designed to allow fine movement of the electrodes during chronic recording.

After the completion of behavioral training, each rat was anesthetized with isoflurane (0.5–3%), and the microdrive with electrodes was chronically fixed to the skull above the right hippocampal CA1 region (AP 3.8, ML 2.0, DV 1.0). The craniotomy was filled with white petrolatum to a level slightly above the point that the tetrodes exited the skull surface. After the supports of the microdrive and cannulas were coated with a thin film of white petrolatum, the entire assembly was embedded in the dental cement on the skull surface. There was a recovery period of ∼1 week following surgery. We lowered the electrodes post-surgery to obtain stable long-term recording. Head stages containing 24 field-effect transistors (Toshiba, Tokyo, Japan) that had been set as source followers were used to connect a 24-channel plastic connector that had been cemented to the animal’s head with preamplifiers.

Multi-neuronal activities and LFPs were amplified and filtered (0.5–10 kHz for spiking activities and 0.5–300 Hz for LFP activity) and recorded at 20 kHz on a custom-made PC with a 24 channel A/D converter (16-bit resolution; Contec Co. Ltd., Osaka, Japan).

### Spike Sorting and Classification of Neurons

Details of spike sorting were previously reported (Takahashi et al., 2003a, b; Sakurai and Takahashi, 2006). Recorded spike trains were sorted to isolate the individual neuronal activities by a method of independent component analysis (ICA) and k-means clustering called ICsort (Takahashi et al., 2003a, b). After spike sorting, the isolation quality was visually inspected in the first to third principal components feature spaces. Only neurons that had interspike intervals >2 ms were analyzed. We identified pyramidal cells based on their wide spike shape (mean width >0.25 ms) and low average firing rate (<5 Hz; Ego-Stengel and Wilson, 2007).

### Statistical Analysis of Spike Data

We used data in correct trials obtained from long fixation trials to examine the temporal modulation of neuronal activity. We consi dered neurons that exhibited a significant difference (*p* < 0.05, Friedman test) in the firing rate over three consecutive non-overlapping periods (1 s each, from 0 to 3 s of the long fixation period) to be temporally modulated neurons (Xu et al., 2014). Spike trains during the long fixation period were smoothed by convolution with a Gaussian kernel (σ = 2 ms) to obtain a spike density function for peak detection.

### Analysis of Local Field Potentials

We used data in correct trials obtained from long fixation trials to examine the temporal modulation of LFP. Because the rats could not discriminate the short and long trial types during the first second, the first 1 s of the long fixation period can be thought to have the same information as the first 1 s of the short fixation period for the rats. We used the Chronux toolbox (Mitra and Bokil, 2008; http://www.chronux.org) and custom-written programs in MATLAB (MathWorks) for multi-taper Fourier analysis. The Chronux function (mtspecgramc.m) was used with the following parameters: 1 s window size, 50 ms time step, time-bandwidth product of 2, and taper count of 3. Before analysis, all LFP data were removed of their direct current offsets, slowly changing components, and 60 Hz line noise by using the locdetrend and rmlinese functions.

### Analysis of Spike-Theta Phase Relationship

To analyze the relationship between spikes and LFPs, we used the Circular Statistics Toolbox (Berens, 2009) available for MATLAB. We used the Rayleigh test to determine whether the distribution of spike phases was uniform (*p* < 0.05).

### Analysis of Theta Power-Reaction Time Relationship

To analyze the relationship between theta power and reaction time (RT), we calculated the Spearman’s rank correlation coefficient between z-transformed theta power during the first second of the fixation and z-transformed RT in the long and short fixation trials, respectively. We used the data which were normalized by each rat.

### Comparison with Error Trials

We compared activities of temporally modulated neurons and power of theta oscillation between the correct and choice error trials. We divided temporally modulated neurons into two groups by peak timings of firing rates: the first group included neurons with the peak of firing rate during the first second of the fixation period and the second group included neurons with the peak between 1 and 3 s of the fixation period. We used the Mann–Whitney *U*-test to compare the firing rate between the correct and error trials for each neuron. In the first group of neurons, we used the data of firing rates occurring between 0 and 1 s of the fixation in both short and long fixation trials for analysis. For the second group of neurons, we used the data of firing rates occurring between 1 and 3 s of the fixation in only long fixation trials. To compare the power of theta oscillation, we used paired *t*-test to analyze the means of theta power of each animal during the first second of the fixation, using data obtained from both the short and long fixation trials.

### Histology

After the experiment was completed, rats were deeply anesthetized with an overdose of sodium pentobarbital (120 mg/kg) before being perfused and fixed with 10% buffered formalin solution. Following fixation, the brain was sectioned in 50-μm intervals, and the locations of the electrode tips and tracks in the brains were identified with the aid of a stereotaxic atlas (Paxinos and Watson, 2009).

## Results

### Behavior

**FIGURE 2:**
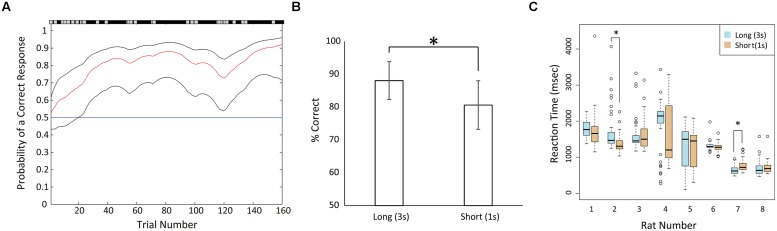
**Behavioral performance in recording sessions**. **(A)** An example of learning process (rat #7) in the first training session of the duration discrimination task. The red line shows the learning curve estimates as a function of trial number and black lines shows upper and lower confidence intervals (95%) for this probability (Smith et al., 2004). The correct and incorrect responses in trials are shown by black and gray marks respectively above the panel. The blue horizontal line shows the chance level. In this example, the rat started learning at trial 20 (the lower confidence interval surpassed the chance level). **(B)** Mean correct rates in the long and short fixation trials in the recording sessions. Error bars show standard deviations. **(C)** Boxplot of reaction times (RTs) of each rat across the recording sessions. Lines inside the box represent medians. The bottom and top of the box show the first and third quartiles, respectively. Whiskers extend to the most extreme data point that is no more than 1.5 times the interquartile range from the box. Open circles show the values of data points that lie beyond the whiskers. Data in long fixation trials were plotted by blue boxes, and data in short fixation trials were plotted by orange boxes. Asterisks indicate significant differences of *p* < 0.05.

After the shaping, all rats became to be able to continue 3 s of fixation. However, the numbers of shaping sessions varied among the rats. For example, one rat (#8) required 19 sessions for shaping, whereas another rat (#7) required 48 sessions. The rats were then trained in the temporal discrimination task. Periods to learn the temporal discrimination task varied among the rats and were much shorter for the animals than the shaping. For example, rat #7 showed the accuracy rate of 80% in the first training session of the task (**Figure 2A**).

After training, all eight rats demonstrated >70% of correct rate. Therefore, we considered that the animals were able to measure the duration of the fixation period and discriminate between the short and long durations. However, the correct rates of short fixation trials (mean = 80.63%, SD = 7.4) were significantly lower than the rates of long fixation trials (mean = 88.07%, SD = 5.77; **Figure 2B**; paired *t*-test, *t*_(7)_ = 2.8457, *p* < 0.05). To confirm the behavioral difference between the two fixation periods, we compared the RTs of each rat for selecting the right or left hole after the door opened (**Table 1**; **Figure 2C**). However, there was no consistent difference among animals. Six of the eight rats demonstrated no significant difference between the two choices (Mann–Whitney *U*-test). One rat (#2) demonstrated significantly faster responses in the short fixation trials than in the long fixation trials (*p* < 0.001); however, the other rat (#7) demonstrated significantly faster responses in the long fixation trials than in the short fixation trials (*p* < 0.001).

### Temporal Modulation of Neuronal Activities

**FIGURE 3:**
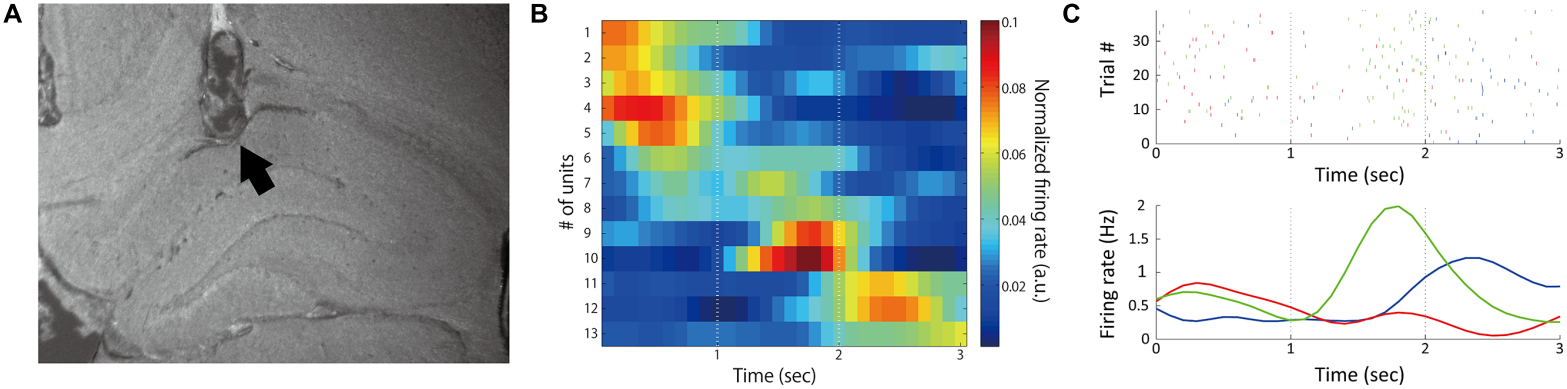
**Temporally modulated neuronal activity. (A)** Sample recording positions verified by histology. The arrow mark indicates the final position of the electrode. **(B)** Normalized firing rates during 3-s fixation periods. Time 0 represents the start of the fixation period. Thirteen neurons recorded from four rats in different sessions were sorted by time of peak firing rate and plotted. Firing rates were normalized by the total firing rates in the time window from 0 to 3 s from the initiation of fixation in each neuron. **(C)** Example activities of three neurons (red: #5, green: #9, blue: #11 in **B**). Upper: raster plots of firing; Lower: peri-stimulus time histograms.

The total number of recorded neurons was 44, of which 33 were putative pyramidal neurons. Thirteen of the 44 neurons (29.5%) showed temporal modulation of neuronal firing (**Figure 3**; **Table 2**). These temporally modulated neurons were recorded from 3 of the 4 rats. **Figure 3B** shows normalized firing rates of the temporally modulated neurons during the long (3 s) fixation period. These neurons fired at different times of the fixation period similar to time cells. **Figure 3C** shows smoothed firing-rate histograms for three examples of neurons that were temporally modulated.

**Table 2:** Number and proportion (%) of neurons exhibiting temporal modulation.

Time modulation		Phase lock
Modulated	13 (29.5%)	2 (4.6%)
	[10]	[1]
Not modulated	31 (70.5%)	5 (11.4%)
	[23]	[5]
Total	44	7 (15.9%)
	[33]	[6]

*The numbers in square brackets are putative pyramidal neurons*.

### Temporal Modulation of Theta Oscillation

**FIGURE 4:**
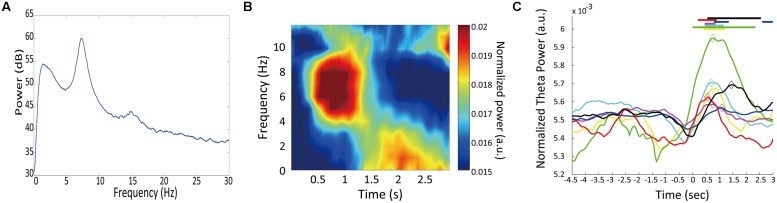
**Temporal modulation of theta oscillations. (A)** A multitaper spectrum example for an LFP recorded from one rat (#4). **(B)** The mean power spectrogram of seven animals. Each animal’s data were normalized by the total power in the time window from 0 to 3 s from fixation start. **(C)** Change in theta power during trials. Normalized theta powers are plotted for individual animals. Horizontal lines at the top represent theta enhancement periods. Diamonds mark the peak theta power for each animal. Each line color corresponds to the “color” in **Table 1**

**Figure 4A** shows an example of power spectrum for LFP recorded in one rat (#5). This spectrum shows strong power in the 4–9 Hz range. This band is known as type 2 theta, which is an oscillation characteristic of rats that are behaviorally immobile or in an alert state (Kramis et al., 1975; Takahashi et al., 2009). To assess temporal modulation of type 2 theta, we calculated a mean power spectrogram of seven animals during the long fixation period (**Figure 4B**). In **Figure 4B**, type 2 theta power shows a transient enhancement around the first 1 s of the fixation period. To examine this power change in more detail, we defined a theta enhancement period as a period in which the theta power was raised above the average power recorded in the ITI period by at least two standard deviations. LFPs from all seven rats had theta enhancement periods at ∼1 s of the fixation period (**Table 1**; **Figure 4C**). On an average, enhancement periods began at 407.14 ms and ended at 1478.57 ms from the start of the fixation period. The average peak points occurred at 871.43 ms (**Table 1**). Only one animal (rat #5) had two theta enhancement periods (**Figure 4C**, blue). However, there is a possibility that the second theta enhancement of this rat was affected by another factor. Because we used a 1 s window to calculate the LFP power, the calculated power after 2.5 s from the initiation of the fixation period may be affected by an event occurring after the fixation, such as movements to release nose poking. Therefore, we chose to exclude this second theta enhancement of rat #5 from the analysis.

**FIGURE 5:**
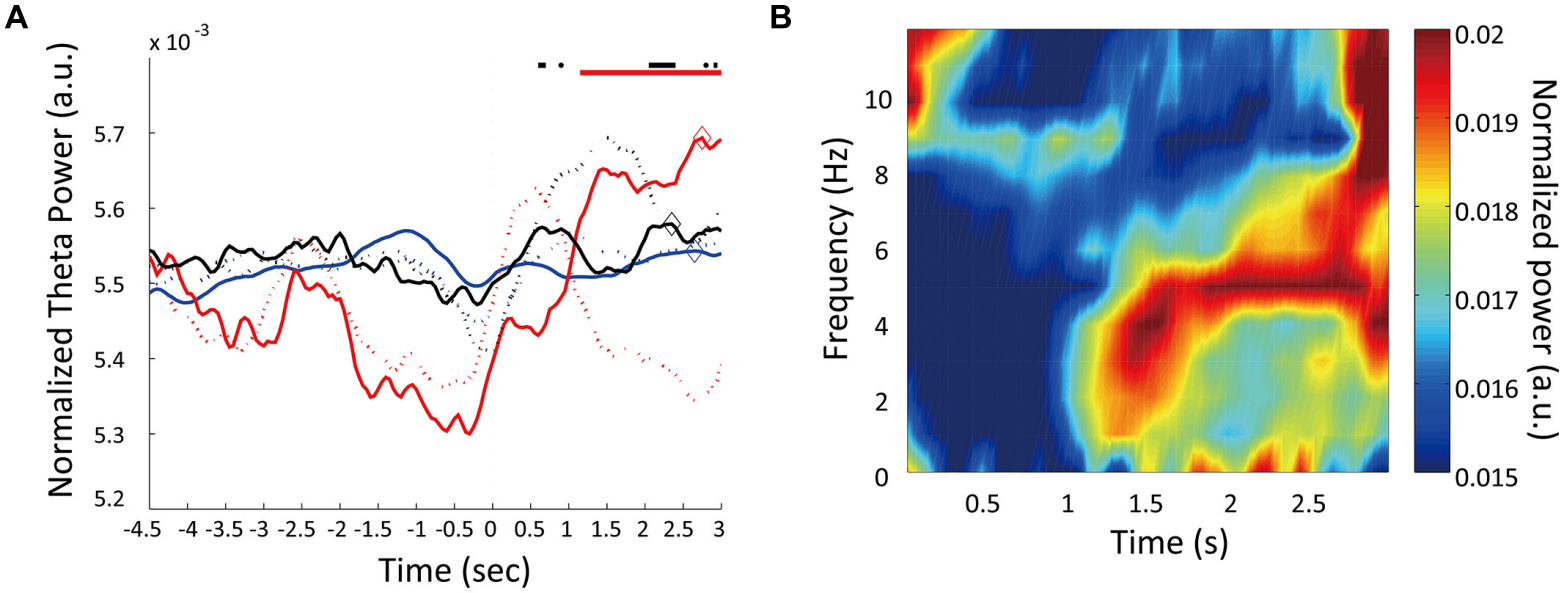
**Temporal modulations of theta oscillations in control condition. (A)** Changes in theta power during control trials. Normalized theta powers are plotted for three animals. Dashed lines are the normalized theta powers during the experimental trials (same as **Figure 4C**). Horizontal lines at the top represent theta enhancement periods. Diamonds mark the peak theta power for each animal. **(B)** The mean power spectrogram of the three animals during control trials. Each animal’s data were normalized by the total power in the time window from 0 to 3 s from fixation start.

To determine whether this theta enhancement simply occurred because of nose-poking movements, we conducted a control experiment. Because the fixation period was 3 s for all trials in the control condition, rats did not have to discriminate the duration of the fixation period; however, the motor movement was the same as in the experimental condition. The recording points of LFP were the same as it were in the experimental condition. In three rats tested in the control condition, theta power exhibited no enhancement at ∼1 s of the fixation period (**Table 1**; **Figure 5**). However, two of the three rats (rat #7, #8) demonstrated theta enhancement at the latter half of the fixation period (**Figure 5A**). The other rats did not show theta enhancement (#5). Moreover, peaks of theta power occurred at 2583.33 ms from the initiation of the fixation period on an average. This indicates that the transient theta power enhancement in experimental condition was not due to the motor movement, and suggested that theta power in the control condition elevated through 3-s fixation period.

### Relationship between Neuronal Activities and LFPs

**FIGURE 6:**
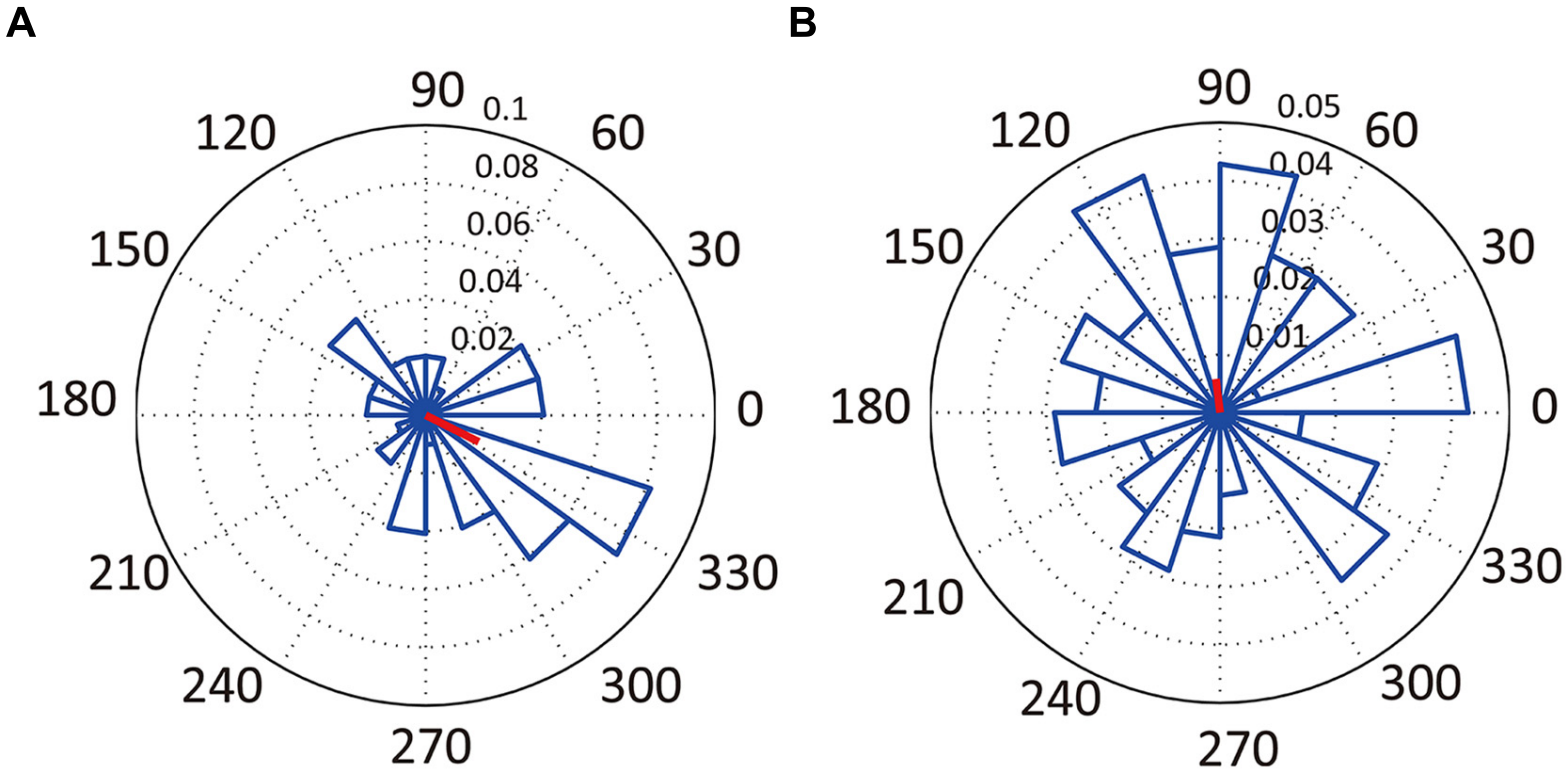
**Relationships between neuronal activity and LFPs.** Examples of angular histograms of significantly phase-locked neuron (**A**, neuron #5 in **Figure 3B**) and of non-significantly phase-locked neuron (**B**, neuron #1 in **Figure 3B**). The radian axis indicates the spiking probability for the corresponding direction. The red lines indicate the direction and magnitude of the mean firing profiles.

Only 15.9% of all recorded neurons were significantly phase-locked to theta oscillations (**Table 2**; **Figure 6**). Of the 13 neurons that were temporally modulated, only two showed significant phase-locking (**Figure 6A**). These results suggest that most of the neurons recorded were not modulated by theta oscillation.

### Relationship between LFPs and Reaction Times

**FIGURE 7:**
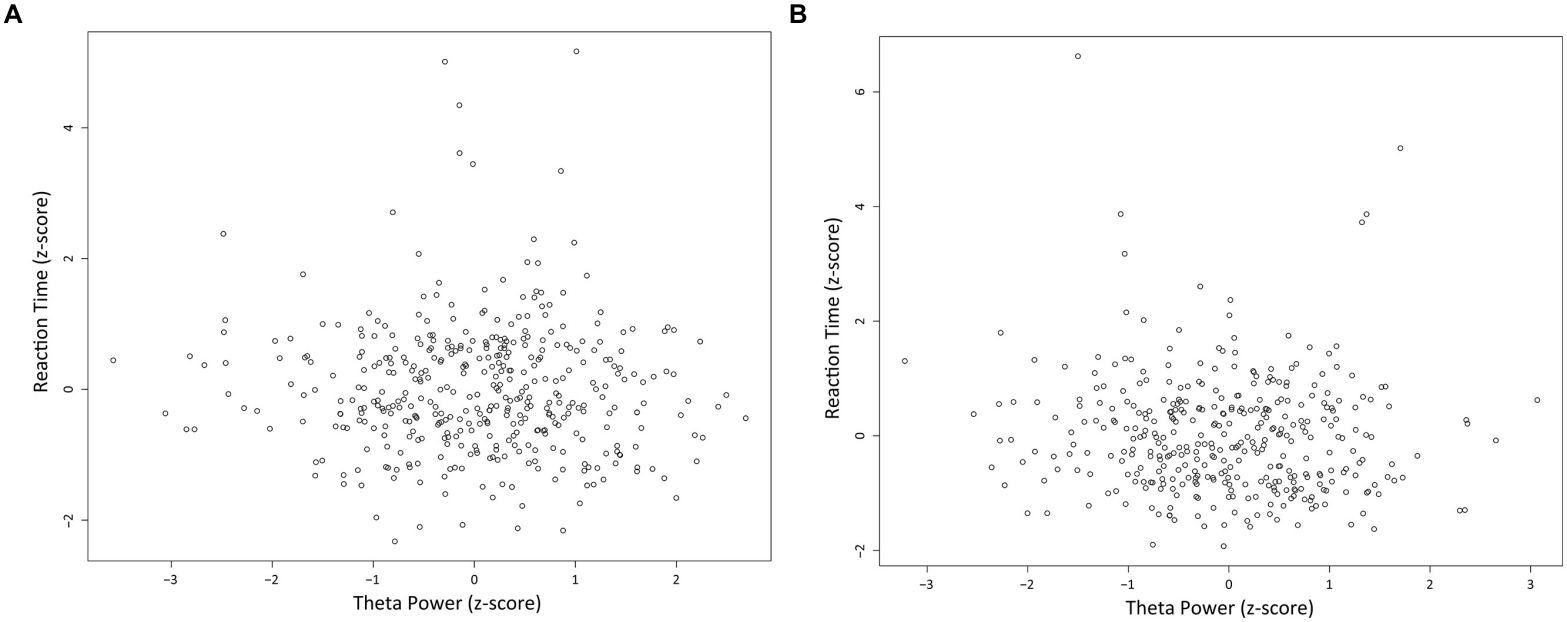
**Relationships between LFPs and reaction times**. Scatter plots of normalized RTs and normalized theta power during the first second of the fixation in long **(A)** and short **(B)** fixation trials.

There was no significant correlation between theta power and RT in both long and short fixation trials. In long fixation trials, Spearman correlation coefficient *r* = -0.029 (**Figure 7A**; *p* = 0.556, *n.s*.). In short fixation trials, Spearman correlation coefficient *r* = -0.084 (**Figure 7B**; *p* = 0.1065, *n.s*.).

### Neuronal Activity and Theta Oscillation in Error Trials

**FIGURE 8:**
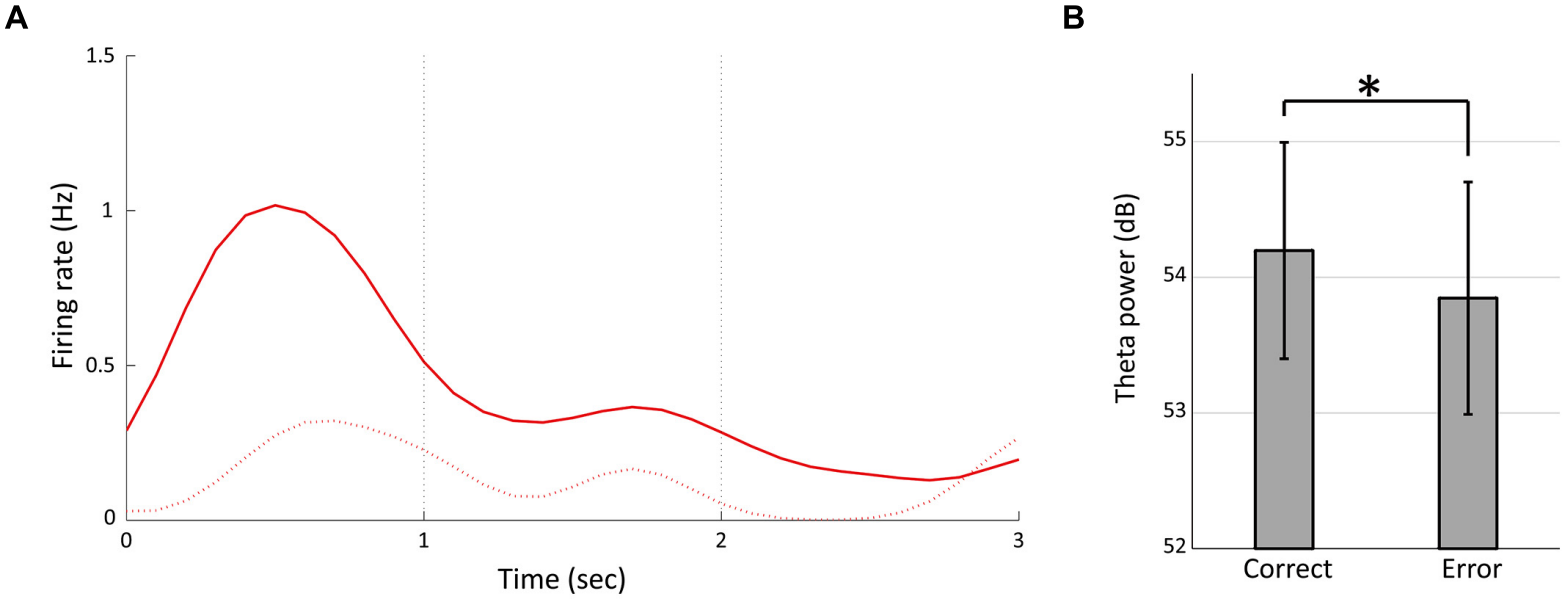
**Temporally modulated neuronal activity and theta power in error trials. (A)** Peri-stimulus time histogram of neuron #5 in **Figure 3** during 3-s fixation periods in the error trials (the dashed line). The solid line represents the firing rate in correct trials (same as **Figure 3C**, Red). **(B)** Mean powers of theta oscillation for the correct and the error trials of each animal. Error bars show standard deviation. Asterisks indicate significant differences of *p* < 0.05.

We divided 13 temporally modulated neurons into two groups by peak timings of firing rates to compare the activities between the correct and choice error trials. The first group comprised neurons having the peak of activity during the first second of the fixation (#1–6, **Figure 3B**) and the second group comprised neurons that had the peak between 1 and 3 s of the fixation (#7–13, **Figure 3B**). We used the data of firing rates between 0 and 1 s of the fixation of both short and long fixation trials for the analysis of the first group and the data of firing rates between 1 and 3 s of the fixation in long fixation trials for analysis of the second group. Two out of the six neurons in the first group and two out of the seven neurons in the second group demonstrated a significant decrease of firing (*p* < 0.05, Mann–Whitney *U*-test) in the error trials. **Figure 8A** shows the example of neuron #5. Moreover, the power of theta oscillation in the error trials was significantly weaker than that in the correct trials (**Figure 8B**; paired *t*-test, *t*_(7)_ = 2.8457, *p* < 0.05).

## Discussion

### Rats Actively Time the Durations of Fixation Period

In our duration discrimination task, all eight rats demonstrated >70% of correct rate. Because there was no cue to choose the correct hole except for the duration of the fixation period in our task, we concluded that all animals were able to measure the duration of the fixation period and discriminate between the short and long durations. However, there were some differences in the behavioral performance between the short and long fixation trials. Animals demonstrated more accurate performance in the long fixation trials than in the short fixation trials (**Figure 2B**), although they demonstrated enough correct answers in the short fixation trials as well (the mean correct rate in short fixation trials was 80.63%). It is possible that long fixation trials were easier for animals because they had more time to make a decision than in short fixation trials. In RTs, we could not find any consistent results among the animals (**Table 1**; **Figure 2C**). Two animals (#2 and #7) had a significant difference in RTs between the short and long fixation trials. **Figure 2C** shows the different response patterns by types of fixation of rat #4, although there was no significant difference in the statistical test. These results suggest that animals had various response strategies in this task.

### Neuronal Activity and Theta Oscillation Changes Support the Concept of Time Cells

Our findings demonstrate that both neuronal spikes and type 2 theta oscillation in the rat hippocampus are temporally modulated during the duration discrimination task. These results support the hypothesis that time cells exist in the hippocampus (MacDonald et al., 2011, 2013; Kraus et al., 2013; Modi et al., 2014), and they operate through sequential firing as if they represent elapsed time. However, the pattern of temporal modulation was different between spikes and theta oscillation, and there was little relationship between them. Therefore, the information processing contributed by spiking activity and theta oscillation may be different and should be examined in more detail.

Though the data presented in **Figure 3** are suggestive of sequential firing, we cannot assert that these neurons were time cells or showed same sequential dynamics in previous studies (Pastalkova et al., 2008; Gill et al., 2011; MacDonald et al., 2011, 2013; Kraus et al., 2013; Modi et al., 2014) because they were recorded from different animals in different recording sessions and the number of recorded neurons is small. Furthermore, we used lower criteria in defining temporally modulated cells than previous studies of time cell (MacDonald et al., 2011, 2013; Kraus et al., 2013). However, four out of the 13 temporally modulated neurons revealed a significant reduction of firing rate in the error trials (**Figure 8A**). It is possible that these neurons have a role in temporal discrimination, although it is unclear whether they correspond to time cells defined in previous studies.

In addition, there was a possibility that behavioral variations during the task affected these neuronal activities. Head positions and head angles were the candidates of such variations. It was reported that neurons of medial prefrontal cortex (mPFC) had a sensitivity to head positions (Euston and McNaughton, 2006; Cowen and McNaughton, 2007). In paticular, Cowen and McNaughton (2007) reported that this phenomenon occured during the delay task in the similar experimental apparatus to that of our task. Even if it was reported that the number of cells which had selectivity to head direction (head direction cells) is small in the hippocampal CA1 (Leutgeb et al., 2000), this possibility should be considerable because mPFC and hippocampus have strong interaction. Furthermore, the effect of head movement should also be considered. Although the head movement was restricted by continued nose-poking during the fixation periods, it was possible that the temporally modulated activity of the neurons, especially the six neurons which had peaks in the initial 1 s of the fixation period (#1–6, **Figure 3B**), were affected by the nose-poke onset movement. Future work is required to examine these possibilities.

### Modulation of Hippocampal Neuronal Activity When Working Memory was not Required

Our results demonstrate that the firing of hippocampal neurons is modulated even when rats perform a simple duration discrimination task in which working memory of task-relevant information is not required (**Figure 3**). In contrast, a previous study found that sequential firing does not occur unless working memory is engaged (Pastalkova et al., 2008). However, our results do not contradict this previous finding because our task may not be regarded as a non-working memory task even though there was no need to hold task-relevant information. Indeed, though our task did not require the animals to retain task-relevant information, there may still be working memory participation in duration discrimination because memory component is indispensable in internal-clock models (Wearden, 1999) and working memory is thought to be an important factor in time perception. The internal clock model comprises three stages (clock, memory, and decision). In this model, short-term memory has an important role during the memory stage. Short-term memory is thought to contain information downloaded from the clock stage, and this information is sent to the decision stage to induce a behavior (Wearden, 1999). It is possible that the working memory participated in our task in this way. Therefore, our results suggest that holding task-relevant information in working memory is not essential for modulating sequential firing of hippocampal neurons, and active time perception, including working memory components, may induce such neuronal firing patterns.

### Transient Enhancement of Type 2 Theta Oscillation in Duration Discrimination Period

When the rats were immobile during the fixation period of the task, LFPs showed distinct 4–9 Hz type 2 theta oscillation (**Figure 4A**). The power of this theta oscillation was mainly increased within the initial 1 s of the fixation period (**Figures 4B,C**). This enhancement was not induced by the nose-poking movement because the enhancement around the first 1 s of the fixation period was not apparent in the rat that performed the control task in which time perception was not required (**Figure 5**) and there was no relationship between the power of theta and movement for choice (**Figure 7**). This enhancement was thought to be linked to temporal discrimination task because this enhancement was observed in all animals (**Table 1**; **Figure 4C**) despite the variability of their response strategies (**Table 1**; **Figure 2C**). The significant decrease of theta power in the error trials supports this speculation (**Figure 8B**).

Although this enhancement of type 2 theta seems to be related to time perception, it is unclear if this enhancement is surely involved in time perception itself or in any other cognitive function such as attention which participates in making correct responses. Because our temporal discrimination task was a two-alternative choice task, we could not completely exclude the possibility that the enhancement of theta was not directly involved in time perception. **Figures 5** and **7**, however, shows that the theta enhancement was related to at least performing temporal discrimination task. More experiments should examine the relationship of the theta enhancement to time perception itself.

In our task, the first 1 s of the fixation period was crucial for discriminating duration because the short fixation period ended at this point (**Figure 1**). Therefore, it is possible that the animals have an active timing mechanism centered around 1 s, and the transient enhancement of theta oscillation contributed to active time perception. Furthermore, it is plausible that the shift of the theta power’s peak timing toward the end of the 3-s fixation period in the control task (**Figure 5A**) occurred through active time perception and the animals expected the door to open after 3 s of fixation. However, how the enhancement of type 2 theta oscillation is involved in active time perception is unclear in this study.

This mechanism may be mediated, at least in part through cholinergic transmission because both type 2 theta oscillation and temporal discrimination functions are related to the cholinergic systems. Anti-cholinergic administration (atropine) has been shown to abolish type 2 theta activity (Kramis et al., 1975) and change time perception (Meck, 1996). However, it is unclear if the cholinergic system involved in time perception is within the hippocampus because the atropine was administered via an intraperitoneal injection in that study, which would affect cholinergic circuits throughout the brain.

### Weak Phase Locking between Neuronal Spikes and Theta Oscillation

MacDonald et al. (2013) demonstrated that the activity of about half of hippocampal pyramidal cells is modulated by the phases of theta oscillations. However, in this study we observed a much smaller number of hippocampal neurons phase-locked to theta oscillations (**Table 2**; **Figure 6**). One possibility for this difference may be that we underestimated the number of phase-locking neurons because of our technical approach, as we recorded spikes and LFPs from different electrodes. Another possibility may be the low load of spatial processing and working memory in this study: the relationship between spikes and theta oscillation in the hippocampus is known to be related to spatial information processing (O’Keefe and Recce, 1993) and working memory (Takahashi et al., 2009), and because there was little load contributed by spatial processing and working memory in our task, it was possible that this interaction was decreased.

### Difference in Temporal Modulation of Neuronal Spikes and Theta Oscillation

Our data indicate a difference in the pattern of temporal modulation between spikes and type 2 theta oscillations. The modulation of activity of many neurons suggests that temporal information is sparsely coded in a population of neurons. This sparse coding is thought to have a role in bridging events as time elapses (MacDonald et al., 2011; Naya and Suzuki, 2011). The previous report (Sakurai, 2002) that hippocampal neurons can simultaneously encode external stimuli and the temporal information inherent in them, but cannot encode temporal information alone, also supports this notion.

Type 2 theta oscillations in the hippocampus exhibited transient peaks at the crucial point in the fixation period of the temporal discrimination task. Type 2 theta oscillations increased in power toward the timing of an expected event (door opening at 1 s of fixation period), and may signal elapsed time. This pattern is similar to neuronal activity that has been observed in striatum (Matell et al., 2003), thalamus (Tanaka, 2007), supplementary motor area (Mita et al., 2009), frontal and supplementary eye fields (Roesch and Olson, 2005), lateral intraparietal cortex (Leon and Shadlen, 2003; Janssen and Shadlen, 2005), and prefrontal cortex (Matell et al., 2003; Roesch and Olson, 2005). The analogous modulation patterns between hippocampal type 2 theta oscillations and neuronal activity in these brain areas suggests a possibility that type 2 theta may participate in interaction between the hippocampus and other brain areas to receive information to perform the task. For example, van der Meer and Redish (2011) reported that neuronal activity in striatum is related to hippocampal theta oscillation.

Prefrontal cortex (PFC) has been shown to have strong interaction with the hippocampus. Previous studies have reported that PFC neurons convey temporal information and that inactivation of PFC impairs time perception (Kim et al., 2013; Xu et al., 2014); therefore, PFC may be related to internal timing mechanisms. Additionally, the interaction between PFC neurons and hippocampal theta oscillation plays an important role in working memory (Jones and Wilson, 2005). Therefore, it is feasible that the same interaction mechanism also contributes to time perception because working memory function is involved in interval timing. In this case, type 2 theta oscillation in hippocampus is assumed as a mechanism to receive temporal information for duration discrimination from PFC.

The results of the present study suggest that there are several distinct temporal information processes mediated in the rat hippocampus. However, because we could not exclude effects of other factors, such as attention, on performance in our duration discrimination task, future works using other behavioral paradigms (for example, peak procedure and temporal bisection task) are required to confirm the present results.

### Is Hippocampus Involved in Time Perception Itself?

Our finding that neuronal activity in the rat hippocampus is temporally modulated seems to conflict with previous studies that claim that the hippocampus is not essential for time perception (Jackson et al., 1998; Kyd et al., 2008; Jacobs et al., 2013). One cause for this discrepancy may be a difference in the time scales tested by the behavioral tasks. We used a time scale of a few seconds, whereas Jacobs et al. (2013) used a time scale of minutes. Additionally, there is a possibility that other brain regions compensated for hippocampal impairment in the other studies, which used lesions to test the role of the hippocampus in time perception.

However, the hypothesis that the hippocampus plays a role in interval timing by interacting with other brain areas does not conflict with previous studies and our results. Furthermore, this hypothesis is consistent with the combined model of time cells (Eichenbaum, 2014). In this model, the mechanisms generating activity in time cells are thought to be a combination of external sources of temporal information and an internal sequential activation in the hippocampus (Eichenbaum, 2014). Our finding that spikes and LFPs have different temporal modulation may support this model, by indicating that theta oscillation in the hippocampus represents flow of information to it.

## Author Contributions

TS, TN, and YS designed the research and wrote the manuscript. TS and TN performed the experiments. ST contributed analytical tools. All of the authors discussed the results and commented on the manuscript.

## Conflict of Interest Statement

The authors declare that the research was conducted in the absence of any commercial or financial relationships that could be construed as a potential conflict of interest.
